# Review of haemovigilance at the Rabat Regional Blood Transfusion Centre in Morocco (2017-2021)

**DOI:** 10.11604/pamj.2024.47.60.42250

**Published:** 2024-02-12

**Authors:** Ilham Lemssahli, Mohammed Benajiba, Abdelkader Belmekki

**Affiliations:** 1 National Blood Transfusion and Haematology Centre, Rabat, Morocco,; 2 Faculty of Medicine and Pharmacy/ Med V University, Rabat, Morocco

**Keywords:** Haemovigilance, transfusion, reactions, safety, Morocco

## Abstract

**Introduction:**

blood transfusion remains an essential therapeutic intervention, but the occurrence of transfusion reactions makes its administration even more complex. Vigilant reporting of such reactions by recipients of blood products is essential for effective haemovigilance. This study aimed to determine the frequency and nature of transfusion reactions.

**Methods:**

conducted over five years (2017-2021) at the Haemovigilance Department of the Rabat Regional Blood Transfusion Centre, this retrospective study exploited incident forms notified by health establishments and data from the regional blood transfusion centre's computer system.

**Results:**

from 1 January 2017 and 31 December 2021, the Rabat Regional Blood Transfusion Centre distributed 435,651 labile blood products to various healthcare establishments, which reported 191 transfusion reactions involving 191 patients. The median age of the patients was 44.3 years, with an overall cumulative incidence of transfusion reactions of 0.44 per 1000 labile blood products delivered. The predominant reactions were non-haemolytic febrile and allergic reactions, accounting for 41.36% and 35.60% respectively. Grade 1 reactions accounted for 87% of all reactions recorded. During the study period, three deaths were recorded, with ABO incompatibility and transfusion-related acute lung injury (TRALI) accounting for two and one case respectively. Transfusion reactions involving erythrocyte components were significantly more frequent than those involving platelet and plasma components.

**Conclusion:**

this study revealed a relatively low incidence of transfusion reactions (0.44%), dominated by non-haemolytic febrile and allergic reactions. Several levels of failure were identified, in particular under-reporting of reactions and inadequate training in transfusion practices and haemovigilance, as well as the need for an effective electronic transfusion reaction reporting system to facilitate reporting and identification of underlying problems and risk factors to improve the quality of transfusion care provided to patients.

## Introduction

Blood transfusion, a critical life-saving intervention, carries inherent potential risks despite its significant benefits. While severe complications are infrequent, acute transfusion reactions (TRs) may occur, necessitating diligent monitoring. These reactions exhibit diverse clinical manifestations influenced by the blood component type, transfusion frequency, and the patient's overall health. A comprehensive understanding of TR frequency and nature is essential for prompt identification and effective management, allowing the implementation of measures to treat and prevent these reactions [1]. Haemovigilance, with its objective of identifying and analysing adverse effects associated with blood transfusion, plays a pivotal role in enhancing the safety of transfusion procedures. Integrated into the quality system for blood transfusion, haemovigilance employs various methodologies, including alert systems, complaint investigations, traceability systems, notification procedures, and practice audits. It is defined as a comprehensive set of surveillance procedures spanning the entire transfusion process, from blood collection to recipient follow-up, collecting information on unexpected effects of labile blood products. The overarching goal is proactive prevention of adverse effects' occurrence and recurrence. Haemovigilance addresses complications in both donors and patients, covering issues in the production line, donor-related complications, near-miss events, retrospective registration of undesired events, and proactive measures with early warnings through a rapid alert system. This comprehensive approach applies to monitoring whole blood and labile blood components, such as red cell concentrates, platelets, and various plasma components like fresh frozen plasma (FFP) [2].

Patients undergoing transfusion commonly undergo physical changes, but most of these changes do not align with the criteria for acute transfusion reactions (ATRs) and can often be ascribed to the patient's underlying diseases and co-morbidities. The overall incidence of ATR is estimated to range from 0.5% to 3%, with variations in the types of ATR depending on factors such as pathophysiology, symptoms, and severity. Diagnosing ATR can be challenging due to the overlap of patient conditions and underlying diseases. Therefore, a comprehensive approach is crucial for the treating physician, particularly after excluding other potential differential diagnoses [3]. In addition, the establishment and effective functioning of a system for information exchange between blood transfusion services and healthcare units are imperative. Such a system ensures seamless communication and collaboration, contributing to enhanced patient care and transfusion safety.

To date, there has been little comprehensive research on blood transfusion complications in Morocco. The objective of this study is to examine transfusion-related reactions reported to the Rabat Regional Blood Transfusion Centre over five years, from 2017 to 2021.

## Methods

**Study setting:** a retrospective evaluation of adverse transfusion reactions spanning five years, from January 1^st^, 2017, to December 31^st^, 2021, was conducted at the Rabat Regional Blood Transfusion Centre in Morocco.

**Study design and context:** Morocco is a North African nation; its capital is Rabat. Morocco is undergoing continuous evolution in its healthcare system. The country boasts modern medical centres across major cities, aiming to provide accessible medical services, including specialized areas like blood transfusion. Despite progress, certain aspects, notably haemovigilance and a comprehensive understanding of blood transfusion complications necessitate further exploration. The aim of studies such as this one, conducted by the Rabat Regional Blood Transfusion Centre from 2017 to 2021, is to shed light on these issues.

In Morocco, the transfusion system is overseen by the National Blood Transfusion and Haematology Centre (NBTHC), comprising 18 regional blood transfusion centres, 14 blood banks, and 24 blood transfusion units. Established through collaboration between the Ministry of Health and the NBTHC, the national haemovigilance system has been a legal requirement since 2005 for all blood transfusion and healthcare establishments [4].

The Rabat Regional Blood Transfusion Centre (RRBTC) holds a pivotal role as a national reference centre, providing labile blood products to over 100 public and private health establishments in the region. Additionally, it actively contributes to the training of healthcare staff in good transfusion practices, transfusion safety, and haemovigilance. The RRBTC utilizes a computerized system (e progesa) for the efficient delivery of blood products to healthcare establishments, ensuring each delivery is accompanied by a detailed delivery note and a transfusion incident form.

**Study population:** the study population included all patients of all genders and age groups admitted to private and public health facilities who ordered blood and experienced a transfusion reaction during the specified period. In addition, all transfusion reactions reported to the RRBTC during the specified period were included in this retrospective study.

### Variables

**Independent variables**

**Period (independent variable):** the duration from January 1^st^, 2017, to December 31^st^, 2021.

**Type of blood component transfused:** categories include red blood cells (RBCs), fresh frozen plasma (FFP), or platelets (PLT).

### Dependent variables

**Transfusion reactions (TRs):** classified by severity grades (grade 1 to 4) based on symptoms reported, laboratory test results, and the type of blood component transfused.

**Frequency of transfusion reactions:** calculated as the percentage of TR cases within each category over the total reported TRs.

**Cumulative incidence of transfusion reactions:** computed annually and overall by establishing the ratio between the number of TRs and the total number of blood products dispensed, expressed per 1,000 units of blood.

**Data collection:** data were collected through transfusion incident forms and the RRBTC information system, detailing medical and transfusion data for each patient. In the event of a transfusion reaction, clinicians completed a transfusion incident form and sent relevant samples to the RRBTC via the healthcare facility's ambulance service. It should be noted that the diagnosis, assessment, and confirmation of the type of TR is carried out following the Centres for Disease Control and Prevention (CDC) criteria (Table 1) [5].

**Table 1:** definition of different types of transfusion reactions following the AABB and CDC criteria

Type of transfusion reaction	Clinical presentation
FNHTR	Fever (≥1°C increase and ≥38.0°C body temperature) within the first four hours of transfusion and/or chills/rigors without any evidence of infection or other conditions causing fever
Allergic reaction	Urticaria, pruritus, rash, oedema, or flushing within the first four hours of transfusion and/or itching sensation without any evidence of other conditions causing allergic reactions
TAD	Acute respiratory distress within the first 24 hours of transfusion without any evidence of other conditions causing similar symptoms, and when TACO and TRALI have been ruled out
TACO	Gallop, jugular venous distension, cough, or dyspnea within the first six hours of transfusion with elevated BNP and CVP with radiologic evidence of pulmonary oedema without any evidence of other conditions causing circulatory overload
TRALI	Respiratory failure, hypotension, fever within the first six hours of transfusion with the evidence of hypoxemia (PaO2/FiO2 ≤300 mm Hg and SaO2
AHTR	Occurs during or within 24 h of cessation of transfusion with new onset of any of the following signs/symptoms: chills/rigors; fever; back/flank pain; hypotension; hematuria; epistaxis; oliguria/anuria; renal failure; disseminated intravascular coagulation (DIC); pain and/or oozing at IV site, as well as positive direct antiglobulin test (DAT) for anti-IgG or anti-C3, positive elution test with alloantibody present on the transfused red blood cells and two or more of the following: elevated LDH; elevated bilirubin; decreased haptoglobin; decreased fibrinogen; haemoglobinemia; hemoglobinuria
Hy TR	Hypotension (≥30 mm Hg drop and ≤80 mm Hg systolic blood pressure) within the first four hours of transfusion without any evidence of other conditions causing hypotension

FNHTR: febrile non-haemolytic transfusion reactions; TAD: transfusion-associated dyspnoea; TACO: transfusion-associated circulatory overload; TRALI: transfusion-related acute lung injury; AHTR: acute hemolytic transfusion reaction

**Sample size:** the sample size for this retrospective study includes all cases involving patients of different sexes and age groups who were admitted to private and public healthcare facilities, ordered blood and experienced a transfusion reaction during the specified period. It is equivalent to the total number of transfusion reactions reported to the RRBTC during the specified period.

**Data analysis:** Microsoft Excel (Office 2007) and STATA 13 were utilized for data management and analysis. Descriptive statistics, including proportions and mean ± 2 SD, were employed. In analysing the data, the following classifications were applied: packed red blood cells (PRBCs), irradiated red blood cells, leukoreduced red blood cells and paediatric red blood cells collectively formed the category called 'red blood cell components'. In addition, the “PLT components” category also apheresis PLT, pooled PLT, and irradiated PLT. In addition, fresh frozen plasma (FFP) is considered a 'plasma component'.

The severity of transfusion reactions was categorised into grades 1 to 4 based on predefined criteria [6]. Patient age was described using the median and interquartile range. The frequency and annual cumulative incidence of transfusion reactions were calculated to assess the prevalence, and the overall cumulative incidence was determined by averaging annual incidences over the entire study period.

**Ethical considerations:** the study was conducted with the approval of the NBTHC director, ensuring patient and donor confidentiality through anonymized data degradation.

## Results

From January 1^st^, 2017, to December 31^st^, 2021, the Rabat Regional Blood Transfusion Centre (RRBTC) distributed a total of 435,651 labile blood products (LBPs) to various public and private healthcare establishments (HEs). The yearly average deliveries amounted to 87,103.2 bags. Among the distributed LBPs, red blood cells (RBCs) constituted the majority at 60.31%, followed by platelets (PLT) at 24.58%, and fresh frozen plasma (FFP) at 15.10%, as detailed in Table 2.

Throughout the 5-year study period, HEs reported a total of 191 transfusion reactions (TRs) involving 191 patients, all of which presented as acute complications. The yearly distribution of TR cases included 42 in 2017, 32 in 2018, 31 in 2019, 48 in 2020, and 38 in 2021 (Table 2). The cumulative annual incidence of TRs examined shows an unstable trend, with a peak in reporting observed in 2020, followed by a slight decrease in 2021. The overall cumulative incidence of TRs calculated is 0.44 per 1,000 LBP delivered (Table 2). In the study, the average age of 191 patients experiencing TRs was 44.3 years, spanning from 7 months to 75 years. Women accounted for more than 50% (56.7%) of the reported complications, while men constituted 43.3% (Table 3).

**Table 2:** statistical data on blood products delivered, number of patients, number and annual incidence of transfusion reactions reported to the RRBTC between 2017-2021, Morocco

Year	RBCs	Platelets	FFP	Total delivered LBPs	Number of patients with TR	TRs reported	Annual incidence TR
2017	51536	19377	12627	83540	42	42	0.50/1000 LBP
2018	55639	20388	13851	89878	32	32	0.35/1000 LBP
2019	56986	21600	16491	92077	31	31	0.34/1000 LBP
2020	48637	22620	11389	82646	48	48	0.58/1000 LBP
2021	49963	23100	14447	87510	38	38	0.43/1000 LBP
Total	262761	107085	65805	435651	191	191	0.44/1000 LBP

RBCs: red blood cells; FFP: fresh frozen plasma; LBPs: labile blood products; TRs: transfusion reactions

**Figure 1:**
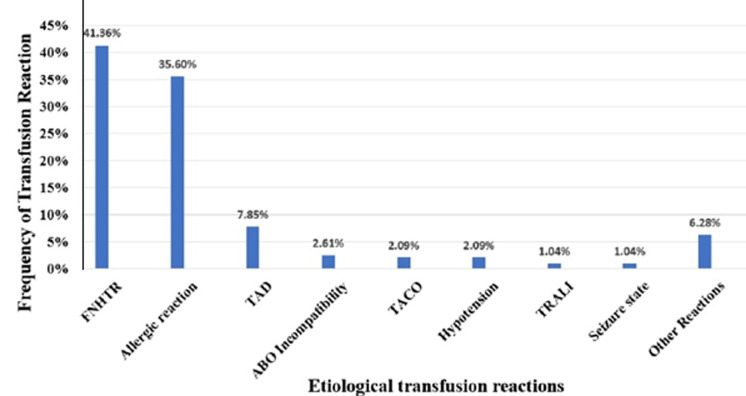
analysis of rates and types of transfusion reactions reported to the Rabat Regional Blood Transfusion Centre from 2017 to 2021, Morocco

During the study period, the healthcare establishments that reported TRs were predominantly sourced from the haemato-oncology departments, the Surgical department, the paediatric hospital, and the maternity hospital. During the study period, transfusion reactions (TRs) were classified as shown in Table 3 and represented in Figure 1: 1) Febrile non-haemolytic transfusion reactions (FNHTR) were the predominant type, accounting for 41.36% (n = 79), followed by allergic reactions (ARs) at 35.60% (n = 68); 2) transfusion-associated dyspnoea was identified in 7.85% of cases (n = 15); 3) transfusion-associated circulatory overload (TACO) was reported in 2.09% (n=4) of cases, in patients aged 18 months, 59 years, 65 years, and 75 years respectively; 4) transfusion-related acute lung injury (TRALI) occurred in 1.04% (two cases), manifested by dyspnoea and agitation. Clinical evaluation revealed bilateral flocculent images on chest X-ray. TRALI was definitively diagnosed in one case of plasma transfusion and was considered a possible TRALI in the second case of platelet transfusion; 5) ABO incompatibility accounted for 2.61% (n=5) of cases among 262,761 red blood cell transfusions, representing an incidence of 0.01/1,000 red blood cells; 6) hypotension occurred in 2.09% of cases (n=4); 7) seizures and loss of consciousness occurred in 1.04% (n=2) following transfusion of fresh frozen plasma. They occurred in patients aged 28 and 55 years respectively. The haemodynamic status of both patients before transfusion was normal (37.2°C, 14/75 mmHg, pulse 80, 37°C and 13/70 mmHg, pulse 90 respectively). Both patients experienced loss of consciousness and explosive clonic movements from the start of the FFP transfusion, requiring immediate interruption of the transfusion. This reaction was not accompanied by cutaneous, respiratory, cardiovascular or gastrointestinal symptoms. Convulsions were associated with a rapid fall in blood pressure. These symptoms disappeared after the transfusion was stopped and the patients were treated symptomatically; 8) other transfusion reactions, non-specific clinical signs and symptoms, were observed in 6,28% (n=12).

**Table 3:** frequency of transfusion reactions recorded in the Haemovigilance Department of the Rabat Regional Blood Transfusion Centre (Morocco) over a 5-year study (2017-2021) based on patient gender and age groups (years)

Patient characteristics					Type of reaction					
Gender	FNHTR	Allergic Reaction	TAD	AHTR(a)	TACO	TRALI	Hy TR	Seizures state	Other RT(b)	Total %
Male	34	29	7	2	2	1	2	0	6	83
Female	45	39	8	3	2	1	2	2	6	108
Total %	79 (41.36)	68 (35.60)	15 (7.85)	05 (2.61)	02 (2.09)	02 (1.04)	04 (2.09)	02 (1.04)	12 (6.28)	191(100%)
Age group 7 months-9 years	14	12	0	3	0	0	0	0	6	35 (17.27)
10-19	12	9	2	0	0	0	0	0	5	28 (14.65)
20-29	10	8	0	0	0	0	0	0	1	19 (9.94)
30-39	7	6	0	0	0	0	2	1	0	16 (8.37)
40-49	8	7	0	0	0	0	0	1	0	16 (8.37)
50-59	8	7	5	1	1	1	2	0	0	25 (13.08)
60-69	5	6	5	1	1	1	0	0	0	19 (9.94)
70-79	15	13	3	0	2	0	0	0	0	33 (17.27)
Total	79	68	15	5	4	2	4	2	12	191(100)

FNHTR: febrile non-haemolytic transfusion reaction; TRALI: transfusion-related acute lung injury; TACO: transfusion-associated circulatory overload; TAD: transfusion-associated dyspnoea; (a)ABO incompatibility; (b) other reactions are non-specific signs and symptoms which not be defined into the certain categories

Among blood and blood components, RBC components were the most frequently implicated in transfusion reactions (81.15%), followed by PLT at 13.08% and FFP at 4.71%. The risk of experiencing transfusion-related reactions per 1000 units of distribution was 0.60, 0.33, and 0.14 for RBC, PLT, and plasma components, respectively. A detailed description of the risk of transfusion-related reactions is provided in Table 4.

**Table 4:** the frequency of transfusion reactions (TRs) related to blood and blood components reported to the RRBT haemovigilance department in Morocco throughout the 5-year study period (2017-2021)

Type of reaction	Blood and blood components involved with the TRs			
	RBCs (a)	PLT(b)	Plasma	Total
FNHTR	70	7	2	79
Allergic reaction	60	7	1	68
TAD	9	5	1	15
ABO incompatibility	5	0	0	5
TACO	1	1	2	4
Hy TR	2	2	0	4
TRALI	0	1	1	2
Other transfusion reaction	8	2	2	12
Total	155	25	9	191
%	81.15%	13.08%	4.71%	100%

FNHTR: febrile non-haemolytic transfusion reaction; TRALI: transfusion-related acute lung injury; TACO: transfusion-associated circulatory overload; TAD: transfusion-associated dyspnoea

**Figure 2:**
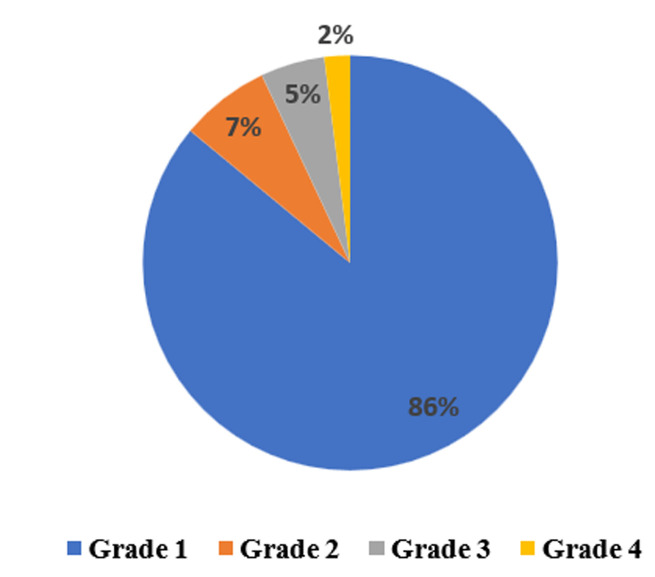
illustration of severity grade of reported transfusion reactions to Rabat Regional Blood Transfusion Centre from 2017 to 2021, Morocco

For the severity classification of transfusion reactions (Figure 2), their examination revealed that the majority of reported cases belonged to grade 1 in 86.38% (n=165). These reactions are characterised by symptoms such as chills, hyperthermia, skin or allergic reactions, digestive symptoms and dyspnoea associated with transfusion. Grade 2 TRs accounted for 7.32% (n=14), mainly involving ABO incompatibilities and hypotension. Grade 3 TRs were observed in 4.71% (n=9), including allergic reactions, seizures, transfusion-associated circulatory overload (TACO) volume overload reactions and transfusion-related acute lung injury (TRALI).

Grade 4 TRs occurred in 1.57% of cases. Notably, three deaths occurred during the study period, two attributed to ABO incompatibility and one to TRALI (Table 5).

**Table 5:** details of grade 4 transfusion reactions notified to Rabat Regional Blood Transfusion Centre from 2017 to 2021, Morocco

Details	Patient’s age	Incriminated LBP	Dysfunctions	Imputability
Case 1: ABO incompatibility	7 months	RBC	Wrong pretransfusion sampling	Certain
Case 2: ABO incompatibility	7 Months	RBC	Wrong pretransfusion sampling	Probable
Case 3: TRALI	65 years	FFP	Unknown	Possible

FNHTR: febrile non-haemolytic transfusion reaction; TRALI: transfusion-related acute lung injury; TAD: transfusion-associated dyspnoea; LBP: labile blood products; FFP: fresh frozen plasma; RBC: red blood cell

## Discussion

The main objectives of a haemovigilance system are to receive and monitor reports from hospitals of adverse events and errors occurring throughout the transfusion chain. The system aims to provide confidential feedback and advice on follow-up actions needed to improve transfusion safety [7]. However, despite the implementation of these surveillance systems, the incidence of non-infectious transfusion reactions (TRs) remains high [1,8].

In the present study, the total number of immediate transfusion reactions reported was 191, out of 435,651 labile blood products (LBP) delivered. The overall cumulative incidence reported was 0.44 per 1000 LBP delivered, indicating a significantly low occurrence compared to incidences reported by other haemovigilance systems. The French National Agency for the Safety of Medicines and Health Products has reported relatively stable rates, in 2021 it was at 3.17 per 1000 LBP transfused [9]. Similarly, Swiss haemovigilance reported that out of 275,343 blood products transfused in 2020, the incidence of adverse transfusion reactions was 7.4 per 1000 LBP transfused [10].

The lower rate observed in this study may not reflect a reduction in transfusion reactions. It is probably associated with the under-reporting of transfusion events in healthcare establishments (HEs) and the lack of accessible data on the amount of blood and blood components administered to patients. Our statistics often refer to the delivery of LBP as we do not have the exact traceability of the products transfused to patients. On the other hand, healthcare professionals may perceive certain transfusion reactions as insignificant or even classify them as “normal”, which contributes to under-reporting. In addition, some reactions go unreported due to a lack of awareness. This finding points to the necessity of implementing targeted training systems and increasing the adoption of haemovigilance systems in healthcare establishments.

Approximately 57% (56.7) of the complications documented in this study occurred in women. Due to the lack of specific data on the number of men and women receiving blood and blood components, a detailed analysis and interpretation of this result remain challenging. Nevertheless, it is noteworthy that maternity hospitals were among the healthcare establishments reporting transfusion reactions (TRs). This observation might be attributed to the multiparous nature of women in these settings, potentially resulting in the presence of circulating antibodies and, consequently, an increased likelihood of transfusion-related reactions [1,11,12].

A notable observation concerning age groups indicated a higher occurrence of transfusion reactions at the extremes of age. The under-20 age group reported a frequency of 31.92%, while the over-60 age group recorded a prevalence of 27.21%. This observation is likely linked to the predominant inclusion of transfusion reaction (TR) reports from haematology-oncology departments, especially those originating from the children's hospital, within the first group. Patients in these departments often undergo multiple transfusions due to conditions such as thalassemia, sickle cell anaemia, and haemopathies, which studies have indicated are associated with increased susceptibility to transfusion reactions [1,11-13]. In the second group, both immediate and delayed complications of transfusion are more common in elderly individuals with diverse comorbidities due to vulnerability factors related to frailty and the risk of multiple transfusions [14].

In our study, the most prevalent transfusion reactions (TRs) were febrile non-haemolytic transfusion reactions (FNHTR) and allergic reactions, constituting 77% of all TRs. This aligns with findings from other haemovigilance systems [10,15,16].

Febrile non-haemolytic transfusion reactions (FNHTR) is frequently associated with red blood cell and platelet transfusions, with plasma transfusions exhibiting a lower frequency [1,17,18]. The occurrence of FNHTR involves two known mechanisms. One is an immune-mediated mechanism in which antibodies (anti-HLA or anti-leukocytes) present in the recipient's plasma react against the leukocytes in the donor components. The other is a non-immune-mediated mechanism facilitated by the presence of Biological Response Modifiers (BRM) accumulating in the donor's plasma during product storage. Febrile non-haemolytic transfusion reactions (FNHTR) stands out as the most prevalent type of transfusion reaction, constituting 29-55% of non-infectious adverse events related to transfusions [19]. The REDS-III report indicates an incidence of non-haemolytic transfusion reactions at 11.3 per 1000 patients [13], translating to approximately 1 per 1632 patients transfused annually, making it the second most common complication of blood transfusion in France [18]. The data collected in our study align with international figures. Typically, symptoms associated with these reactions resolve within 2 to 3 hours, with or without symptomatic treatment [10,15,16]. Research has shown the effectiveness of leukoreduction in reducing the incidence of these reactions. Pre-storage leukoreduction is more effective than post-storage leukoreduction in preventing FNHTR, as it reduces donor antigens and active biological factors released by white blood cells during storage. However, these reactions persist in some patients, likely due to the residual presence of white blood cells and active biological factors [18,20,21].

The incidence of FNHTR reported in this study was relatively high because leukoreduction of RBCs and PLTs was not conducted systematically but rather upon clinicians' request. To address these reactions comprehensively, a systematic implementation of leukoreduction for red blood cells will commence in January 2022. The National Blood Transfusion and Haematology Centre (NBTCH) has decided to replace the previously used triple bags for blood collection with quadruple bags.

**Allergic transfusion reactions:** are typically linked to a soluble allergen carried by the donor's plasma to a sensitized recipient with specific IgE. They account for 17-40% of all transfusion reactions [1,22,23]. The characteristics of the recipient, the product, and the donor are all likely to be involved [17]. ATRs are generally attributed to a soluble allergen brought by the donor's plasma to a recipient who is already sensitised and presents specific IgE.

Clinical manifestations vary in severity, with most cases falling into the mild grade 1 category, often presenting as urticaria, with or without pruritus. Conversely, severe reactions, including anaphylaxis or anaphylactic shock, occur in less than 1% of cases [24]. Establishing a direct causal link between these reactions and a specific labile blood product is challenging, as there is no definitive test available. Diagnosis relies on a combination of suggestive. symptoms, a compatible temporal relationship, and the absence of any other apparent cause. Allergic reactions are more prevalent with platelet and plasma products, with some reports indicating an incidence of 1-4% in response to platelet transfusions. Combined platelet products seem to be associated with even higher rates of allergic transfusion reactions compared to apheresis platelets [8,23,25]. Regarding the involvement of red blood cells (RBCs) in allergic reactions, conflicting findings exist in the literature, with certain studies suggesting RBCs contribute to 0.4% of reactions, while others report a rate of 4.1% comparable to platelets [26].

The findings of our study align with several publications, indicating that allergic transfusion reactions rank as the second most frequent cause of transfusion reactions. These reactions typically manifest as grade 1, with predominant mucocutaneous signs such as urticaria and pruritus, often associated with transfusions of red blood cells and platelets. Interrupting transfusions after the onset of mild allergic reactions is common, and typically, transfusions are resumed only when a different LBP is obtained [10,18,27]. Clinical experience suggests that once symptoms have subsided, transfusion can be resumed with the same unit at a lower rate under continuous monitoring. However, transfusion should be discontinued if symptoms reoccur or if other local skin symptoms develop [20,28]. To mitigate these reactions, certain strategies, such as removing excess plasma from platelets and washing red blood cells and platelet products, have demonstrated efficacy in significantly reducing the incidence of allergic transfusion reactions [20,29]. Despite this, these strategies are not widely implemented in transfusion centres in Morocco.

**Pulmonary complications:** pulmonary complications of transfusion are among the most dangerous and account for the highest number of transfusion-related deaths. Transfused patients are often aged and have significant co-morbidity. Experts reviewing these cases find it difficult to classify them, often due to a lack of essential data [30]. In the present study, the most frequently reported pulmonary manifestations were transfusion-associated dyspnoea (TAD) which appeared during transfusion and subsided after the transfusion was interrupted in patients aged more than 50 years.

The frequency of TAD reported in the present study (7.85%) was higher than that described by other studies [1,3,11,31]. This may be explained by a lack of clinicians' information on pulmonary complications, who often confuse them with other pulmonary complications such as TACO and TRALI.

Transfusion-associated circulatory overload (TACO) is the leading cause of transfusion death in France and the United Kingdom [18,30] and the second leading cause of transfusion death in the United States of America, accounting for 21% of deaths in 2012 [17,32]. Its exact incidence depends on the population studied.

The incidence reported by the French system of haemovigilance is 1/10907 LBP transfused [11]. Other studies have reported a higher incidence ranging from 1% to 5.7% in transfused patients [32-34].

In the present study, TACO was observed in patients aged over 50 years, extreme age is one of the known risk factors for TACO [1,35]. The incidence calculated was 0.01/1000 LBPs, which is still very low compared with international data [17,18,32-34]. The incidence is underestimated due to under-reporting and lack of awareness of TACO among clinicians. Transfusion-associated circulatory overload (TACO) is often confused with the patient's initial pathology and is not considered to be related to the transfusion procedure.

Transfusion-related acute lung injury (TRALI) represents a serious complication that is poorly understood by clinicians [16,36]. TRALI has either immunological or non-immunological origins. The immunological origin is mediated by HLA antibodies (classes I and II) or neutrophils (human neutrophil antigens (HNA)) in the donor plasma that react against the recipient's cells, causing lung damage characterized by neutrophil recruitment and activation. The non-immunological origin is secondary to modifiers of the biological response [20,37-39]. All blood components can trigger TRALI, but plasma-rich components are associated with an increased risk [17]. Transfusion-related acute lung injury (TRALI) is widely underdiagnosed and underappreciated, and its true incidence is unknown, as transfusion of FFP from female donors is widely used.

In this study, the incidence of TRALI was approximately 0.005/1000 LBPs. A fatal case of TRALI was reported in a 65-year-old patient following a transfusion of fresh frozen plasma. The incidence of TRALI is low, uncertain, and certainly underestimated, especially considering that plasma from female donors is commonly used in Morocco.

**ABO Incompatibility:** ABO incompatibilities transfusion reactions are acute and serious haemolytic transfusion reactions that represent a major risk in blood transfusion and are directly related to the volume of blood transfused. They are attributable to an immunological conflict between the antigens present on the membranes of transfused red blood cells and the antibodies present in the patient's plasma [15-18]. The risk of ABO incompatible transfusion has been estimated to be three times higher than the combined risk of transfusion transmission of hepatitis B, hepatitis C and HIV [40]. French haemovigilance estimates their incidence at 1/184465 blood products transfused [18].

The UK Serious Hazards of Transfusion haemovigilance scheme (SHOT) has reported that 249 (13%) of the 1832 accidents were ABO incompatibilities transfusion reactions. The risk of error was estimated at 1/15000 blood products distributed, and 1/100000 ABO incompatible transfusions performed. In 30% of cases, major morbidity occurs requiring intensive care or renal dialysis, and 5-10% of episodes contribute to the patient's death [16,41].

In the present study, these reactions accounted for 2.61%, corresponding to an incidence of 0.01/1000LBP transfused, including 02 reported deaths. The ABO incompatibilities were not the result of laboratory errors, but, as in the majority of studies, the errors occurred at the hospital level. They resulted from non-compliance with standard transfusion procedures. The main causes are errors identified at different links in the transfusion chain: error in identifying the recipient, compatibility test not performed, incorrect allocation of blood units, and final check at the patient's bed not performed or performed incorrectly. The final check at the patient's bed, the last safety lock performed correctly, can prevent ABO incompatible transfusions [15-18,20,27,40]. The incidence reported in this study is low, underestimated, and is only the tip of a much larger iceberg. Various measures have been recommended to prevent these reactions and avoid the development of other fatal ABO incompatibility reactions, including transfusion training, skills assessment, pre-transfusion safety checks using a checklist placed next to the patient to avoid incorrect transfusions. In our context, training sessions dedicated to paramedical staff have been stepped up, focusing on the final steps in the transfusion process at the patient's bedside, the importance of complying with good transfusion practice, and the need to report such incidents.

**Hypotensive transfusion reactions:** these reactions are believed to be associated with an increase in vasoactive bradykinin (BK) and its active metabolites (des-Arg9-BK) as a result of increased production and/or decreased metabolism of des-Arg9-BK [20,42,43]. Some retrospective studies have reported rates ranging from 0.078 to approximately 10 per 1000 transfusions [20]. In the current study, the rate of hypotensive transfusion reactions was 2.09%, their incidence is estimated at 0.01/1000 LBPs transfused, which remains relatively low compared with the data published in the literature. These reactions are probably underestimated as they are usually temporary and considered ordinary reactions by clinicians, which generally resolve rapidly without specific treatment. Consequently, this study does not allow any definitive conclusions to be drawn about the actual frequency of these reactions or the products involved.

**Seizure state:** the presence of convulsive activity or refractory shock during a blood transfusion with loss of consciousness is generally referred to as an anaphylactic reaction. It is characterised clinically by the sudden onset of signs and symptoms, including skin manifestations in the majority of anaphylactic reactions [44]. In this study, 2 cases of seizures were considered to be secondary to profound hypotension resulting in decreased cerebral perfusion and hypoxia. Neither case was classified as anaphylactic shock and no investigation in this respect was undertaken, especially as the signs disappeared after the transfusion was stopped.

**Other unclassifiable transfusion reactions:** in our study, it proved impossible to classify 6.28% of transfusion reactions, for various reasons. This finding is consistent with a prevalent trend in haemovigilance reports, where unclassifiable reactions are recurrent, highlighting the need for those involved in the transfusion process to identify deficiencies [16]. Our study identified several factors that contribute to unclassifiable reactions, including late notification preventing a thorough investigation, the absence of adequate sampling, either of the patient or the LBP potentially involved; and a lack of clinical information on the transfusion information sheet, leading to gaps in investigation and inconclusive results. To promote the standardisation of procedures, we strongly advocate awareness sessions and ongoing training for all stakeholders involved in transfusion.

**Grade of transfusion reaction severity:** this study revealed a significant association between red blood cell (RBC) components and transfusion-related reactions, aligning with findings from the majority of previous studies [1,11,45,46]. The increased utilization of RBC components within healthcare establishments settings might account for this observed pattern. Overall, disparities in the quality of blood components, patient characteristics in receipt of blood products, and the frequency of transfusions could contribute to varying rates of transfusion reactions across red blood cells (RBC), platelets (PLT), and plasma components [1,47].

Grade 1 transfusion reactions were the most frequent, followed by grade 2. These results are in line with those of other studies: grade 1 accounted for 92% of accidents in French haemovigilance reports [9] and in other Moroccan studies [15].

Our study was limited by the absence of data on delayed adverse events, which include alloimmunisation and infectious diseases. Near-miss events are not considered, even though they are recognised as the hidden faces of blood transfusion errors. In addition, the significant number of undefined adverse reactions and cases where patients die by mistake during a transfusion conceal numerous anomalies and non-compliances. To meet these challenges, we need to investigate further and identify problems within our system to improve the quality of blood transfusions.

## Conclusion

In this study, the incidence of transfusion reactions was relatively low, around 0.44%. The majority of these reactions were non-haemolytic febrile reactions and allergic reactions. Adopting quadruple bags during blood collection and using leukoreduced blood components may help to reduce these reactions. The results of the study not only provide valuable data on transfusion reactions but also highlight the inadequacy and considerable variability of our results over the years. Different levels of failure were highlighted, including under-reporting of transfusion reactions, malfunctions in various aspects of the transfusion process often attributed to human error, and inadequate haemovigilance training. Effective diagnosis of a transfusion reaction requires a thorough understanding of investigation procedures and close collaboration between all participants in the transfusion chain. Our adverse reaction reporting system, which is currently paper-based, could benefit considerably from the adoption of an electronic system. This transition could improve traceability, increase reporting rates, and ensure the security of data collection. Corrective action should prioritise regular training sessions and increased awareness among healthcare staff of the central role of transfusion reaction reporting in the continuous improvement of transfusion practices. However, substantial progress underlines the benefits of a transfusion reaction reporting system.

### What is known about this topic

- *Haemovigilance systems are an essential source of data for assessing adverse events and reactions in the field of blood transfusion;*
- *The collection, analysis and evaluation of hemovigilance data help to identify patterns, trends and potential risk factors associated with blood transfusions;*
- *Hemovigilance data can be used in education and training programs for healthcare professionals involved in blood transfusion*.

### What this study adds

- *The study shows that transfusion reaction notification rates are very low due to under-notification, fatal transfusions are mainly due to errors at the hospital level, knowledge of certain transfusion reactions is insufficient, and there is a need to organize training sessions for users of blood products;*
- *Despite its implementation in 2005, Morocco's haemovigilance system remains ineffective;*
- *Further efforts are needed to train healthcare professionals and raise their awareness of the importance of hemovigilance and transfusion safety*.
